# Interaction of Mg Alloy with PLA Electrospun Nanofibers Coating in Understanding Changes of Corrosion, Wettability, and pH

**DOI:** 10.3390/nano12081369

**Published:** 2022-04-16

**Authors:** Manuela Elena Voicu, Ioana Demetrescu, Andrei Dorobantu, Marius Enachescu, George-Octavian Buica, Daniela Ionita

**Affiliations:** 1 Department of General Chemistry, Faculty of Applied Chemistry and Material Science, University Politehnica of Bucharest, 313 Splaiul Independentei, 060042 Bucharest, Romania; manuela_elena.voicu@upb.ro (M.E.V.); ioana.demetrescu@upb.ro (I.D.); 2 Academy of Romanian Scientists, 3 Ilfov Street, 050044 Bucharest, Romania; 3 Center for Surface Science and Nanotechnology, University Politehnica of Bucharest, 060042 Bucharest, Romania; andrei.dorobantu@cssnt-upb.ro

**Keywords:** magnesium alloy, PLA nanofibers, coating, biodegradable property, electrochemical and morphology characterization

## Abstract

A modified biodegradable magnesium alloy (AZ31, 96 wt% Mg, 3 wt% Al, and 1 wt% Zn) with polylactic acid (PLA) nanofibers was obtained by the electrospinning technique. The presence of PLA nanofibers was evidenced using Fourier transform infrared spectroscopy (FT-IR) and using an scanning electronic microscope (SEM) equipped with an energy dispersive X-ray spectroscopy (EDX) module. The degradation behavior of an uncoated Mg alloy and a Mg alloy coated with PLA was evaluated through hydrogen evolution, pH, and electrochemical measurements in simulated body fluid. Contact angle measurements showed a shift from hydrophilic towards the hydrophobic character of the alloy after its coating with PLA nanofibers. Furthermore, the electrochemical measurement results show that the Mg based alloy coated with PLA inhibits hydrogen evolution, thus being less prone to degradation. The aim of this research is not only to reduce the corrosion rate of Mg alloy and to improve its properties with the help of polylactic acid coating, but also to provide a study to understand the hydrophilic–hydrophobic balance of biodegradable magnesium based on surface energy investigations. Taking into account corrosion rate, wettability, and pH changes, an empiric model of the interaction of Mg alloy with PLA nanofibers is proposed.

## 1. Introduction

Stainless steel, CoCr, and Ti alloys, coated or uncoated, have traditionally been employed for medical device implants in surgery due to their improved mechanical and corrosion properties as well as high biocompatibility [1,2,3]. Because such implants are non-biodegradable, they must frequently be removed from the human body by a further operation after healing. This implies new risks of infection, although the implants are specially coated and most of them present a good antibacterial effect [4,5]. To solve this issue, introducing directly temporary implants that degrade themselves in the human body was one of the strategies permitting the reduction of patient time spent in hospitals. The degradable metals for biomedical application need to restore tissue function having properties close to human bone [6,7]. In this context, simultaneously with the increasing number of secondary operations, a growing interest in biodegradable materials has developed [8]. Although Zn, Mg, and Fe are among the most basic biodegradable metals, Mg not only possesses an elastic modulus similar to that of human bone [9], but it also provides crucial nutrition for the human body [10]. The primary drawback of biodegradable magnesium is that it dissolves rapidly in the human body and has a high corrosion rate at pH 7.4–7.6. As a result, hydrogen is released, which is detrimental to the implant as well as the patient [11,12].

Thus, as a relatively recent concept for the improvement of Mg corrodible metallic biomaterials by alloying and modifying the surface with the different coatings developed, it has resulted in the development of a new generation of bioactive biomaterials with potential multifunctional capabilities [13,14,15]. Alloying is an effective procedure that changes the structure and properties of a metal. Therefore, to prevent tissue damage and to ensure faster healing, magnesium alloyed with various metals are used, such as aluminum (Al), calcium (Ca), and zinc (Zn) [16]. For example, alloying magnesium with aluminum has a number of benefits, including improved biodegradability, mechanical characteristics or corrosion resistance [17,18].

Another method for improving the qualities of magnesium materials is to apply surface changes such as coatings, which are less expensive and more widely used [19]. Among the different materials used for coating biodegradable magnesium alloys, synthetic polymers seem to be the most typical, as they lead to an increase in electrochemical stability due to the insulating properties that ensure the absence of galvanic corrosion between coatings and substrate [20]. Thus, in recent years, biocompatible polymers such as poly(lactide-co-glycolic) acid, poly(glycolic acid), collagen, polycaprolactone, chitosan, polylactic acid (PLA), fibrin, alginate, and other well-known biocompatible polymers have been investigated and tested as coatings [21].

In the case of a biodegradable implant, a quick corrosion process is crucial since the implant must preserve its mechanical stability for a specified period of time before the bone heals entirely [22].

Therefore, coating the Mg alloy with polylactic acid (PLA) helps to slow down the dissolution of the alloy and maintain a balance between bone regeneration and implant resorption [23].

Thus, the aim of this research is not only to reduce the corrosion rate of Mg alloy and to improve its properties with the help of polylactic acid coating, but also to provide a study to understand the hydrophilic–hydrophobic balance of biodegradable magnesium based on surface energy investigations. The understanding of such changes is based on more knowledge about the Mg alloy interaction with PLA electrospun nanofibers. The interaction mechanism of the Mg alloy with a PLA nanofiber coating could explain the increase of the surface’s hydrophobic character being an important novelty for more potential applications of biodegradable Mg alloys, such as platelet adhesion decrease in thrombosis or drug release. As an expression of novelty, an empiric model of interaction is proposed, based on experimental data.

## 2. Materials and Methods

### 2.1. Reagents

AZ31 alloy (96 wt% Mg, 3 wt% Al, and 1 wt% Zn) from Alfa-Aesar, Thermo Fisher, Kandel Germany, PLA (granules 3 mm—GoodFellow, Huntingdon, UK), chloroform (99%—Carl Roth, Germany) and N, N-dimethylformamide (99.9% HPLC grade—Alfa-Aesar, Haverhill, MA, USA) were used to prepare the solution for nanofiber deposition. All the salts required to prepare simulated body fluid solution (SBF) were from Sigma-Aldrich (analytical reagent grade). Aqueous solutions were prepared using Type I ultrapure water obtained from a water purification system (Millipore Direct Q 3UV from Merck, Molsheim, France, 18.2 MΩcm^−1^).

### 2.2. Equipment

PLA nanofiber deposition by electrospinning was achieved using a high-power source (PS/EJ30P20, Glassman High Voltage, Inc., High Bridge, NJ, USA) connected to a pump (Legato 180, KD Scientific, Holliston, MA, USA).

A Contact Angle Meter–KSV Instruments CAM100 was used to measure the contact angle by dripping 3 drops of distilled water, ethylene glycol, and diiodomethane on different areas of each sample of uncoated Mg alloy, as well as Mg alloy coated with PLA nanofibers (Mg alloy-PLA). Each determination was repeated 3 times for the three liquids.

FT-IR spectra were recorded on a Perkin-Elmer Spectrum 100 spectrophotometer.

The SEM-EDX analysis was performed using a Hitachi SU 8230 scanning electron microscope equipped with an Oxford EDX detector-analyzer.

The electrochemical measurements were performed in a three-electrode electrochemical cell connected to a potentiostat–galvanostat from AutoLAB PGSTAT100N (Metrohm Autolab, Barendrecht, The Netherlands). The working electrodes were Mg alloy and Mg alloy coated with PLA nanofibers (6 mm in diameter–controlled surface). The counter electrode was a platinum (Pt) wire, while the reference electrode was an Ag/AgCl electrode. At a rate of 2 mV/s, potentiodynamic voltammetry was performed vs. open circuit potential (OCP). Electrochemical impedance spectroscopy (EIS) was conducted at OCP with a 10 mV applied signal and frequencies ranging from 10^4^ to 10^−1^ Hz. The readings were taken at varied intervals of time.

### 2.3. Procedures

#### 2.3.1. Preparation of AZ31 Biodegradable Alloy

After gradually polishing up to 1200 grit with SiC paper, samples of AZ31 alloy with dimensions of 20 mm × 20 mm × 1 mm were used. The samples were then cleaned and degreased in the ultrasonic bath with ultrapure water (10 min) and ethyl alcohol (10 min) before being dried in air.

#### 2.3.2. Preparation of Simulated Body Fluid Solution (SBF)

The electrolyte utilized to characterize the Mg alloy was simulated body fluid (SBF), which was prepared according to Kokubo’s recipe. Table 1 provides the composition of the SBF.

#### 2.3.3. Deposition of PLA Nanofibers on AZ31 Alloy

PLA nanofibers were deposited by the electrospinning technique using a solution obtained from 0.173 g PLA granules dissolved in a mixture of 1.153 mL chloroform (CHCl_3_) and 0.77 mL N, N-dimethylformamide (DMF) under magnetic stirring and alternating with the ultrasonic bath until the complete dissolution of the PLA and obtaining a homogeneous solution. The PLA solution was transferred to a 1 mL plastic syringe with a needle, which was then connected to a pump having a flow rate of 0.5 mL/h. The PLA solution was deposited on the Mg alloy samples using a constant voltage of 20 kV. The deposition time was 1 h, and the distance between the collector and the needle of the syringe containing the PLA solution was 15 cm.

#### 2.3.4. Hydrogen Evolution

**Scheme 1:**
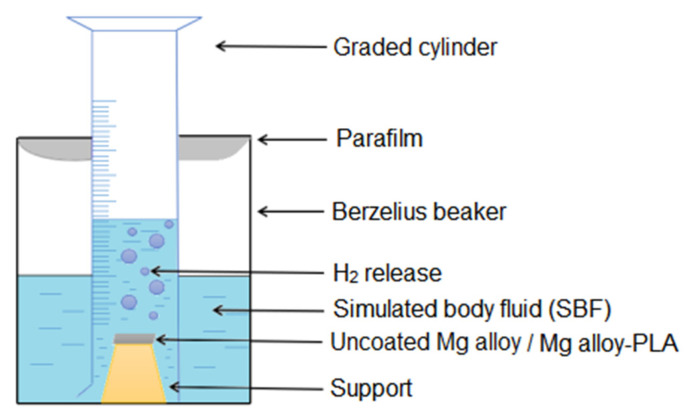
Schematic illustration for volume measurement of H_2_ evolution.

Hydrogen evolution for uncoated Mg alloy and Mg alloy-PLA samples was evaluated in SBF of pH 7.4, at different time intervals (1 h, 3 h, 8 h, 24 h, 72 h, 168 h, 240 h, 408 h, and 504 h) using the equipment shown in Scheme 1. It consisted of a beaker inside which a plastic support containing the sample of uncoated Mg alloy or, respectively, Mg alloy-PLA, was fixed. A graded cylinder containing SBF solution was placed upside down on the beaker to determine the volume of hydrogen by changing the level of solution during the experiment, according to ASTM-G31-72 protocol (ratio surface-solution 1 cm^2^: 30 mL) [25]. The whole setup was covered with parafilm. The volume of hydrogen released is proportional to the amount of dissolved magnesium alloy. Thus, the volume of H_2_ collected is converted into mass loss, 1 mL H_2_ released corresponds to 0.00108 g of decomposed Mg alloy. The amount of H_2_ released is unaffected by the corrosion products generated on the Mg surface. This experiment is based on reaction (1), according to which hydrogen is produced during the corrosion process [26].

Mg(s) + 2H_2_O(aq) → Mg(OH)_2_(s) + H_2_(g) (1)

Corrosion rate (CR, mm/year) was calculated based on the evolution of the volume of H_2_ released using the following Equation (2) [26]:

CR (mm/year) = k rate of H_2_ evolution (mL/cm^2^/day) (2)

where: 

k—constant, calculated with: $k=\left(\frac{365\;\mathrm{days}}{\rho \cdot \;1\;\mathrm{year}}\cdot \frac{1\;g}{1000\;\mathrm{mm}}\cdot \frac{1\;\mathrm{mm}}{1\;\mathrm{cm}}\right)$. ρ—density, AZ31 = 1.82 g/cm^3^.

## 3. Results and Discussion

### 3.1. Polymer Coating Characterization

#### 3.1.1. FT-IR Analysis of Coating

**Figure 1:**
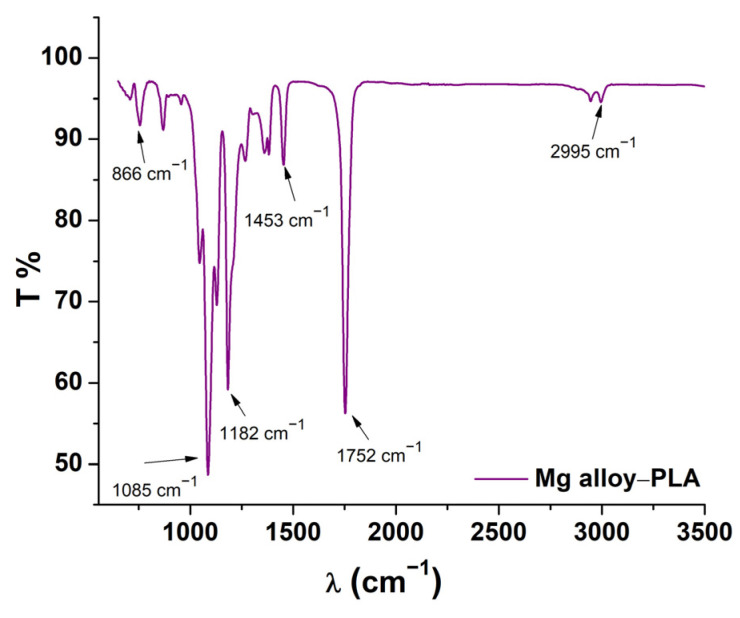
FT-IR spectrum of polylactic acid.

Following FT-IR analysis, the proof of the deposition of PLA on the Mg alloy is shown in Figure 1, which presented characteristic IR bands of polylactic acid. The observed bands, the carbonyl group (C=O) at the wavelength 1752 cm^−1^, the C-O-C group at 1085 and 1182 cm^−1^, the C-C group at 866 cm^−1^, the CH_3_ group at 1452 cm^−1^, and the characteristic bands C-H (CH_3_) group at 2945–2995 cm^−1^, indicated that the layer deposited on the surface of the Mg alloy was composed mainly of PLA [27,28]. The absence of signals in the 700–830 area indicated that the chloroform (CHCl_3_) solvent used to dissolve the PLA granules had been completely removed.

Using Equation (3), the thickness of the PLA coating was determined by measuring the weight and surface area of the Mg alloy before and after PLA coating [29]:

(3) $$\mathrm{Thickness}\;\left(\mu m\right)=\frac{\mathrm{weight}\;\mathrm{gain}}{\mathrm{density}\;\mathrm{surface}\;\mathrm{area}}\cdot 10^{4}$$

The density of the magnesium alloy was 1.82 g/mL (provided by the manufacturer). The value of the thickness of PLA was 2.8 µm. It is known that a coating which is too thin is ineffective in reducing water permeation. In contrast, if the coating is too thick it may raise concerns about the inflammatory reactions associated with polymer degradation products.

#### 3.1.2. Morphology and Elemental Composition

**Figure 2:**
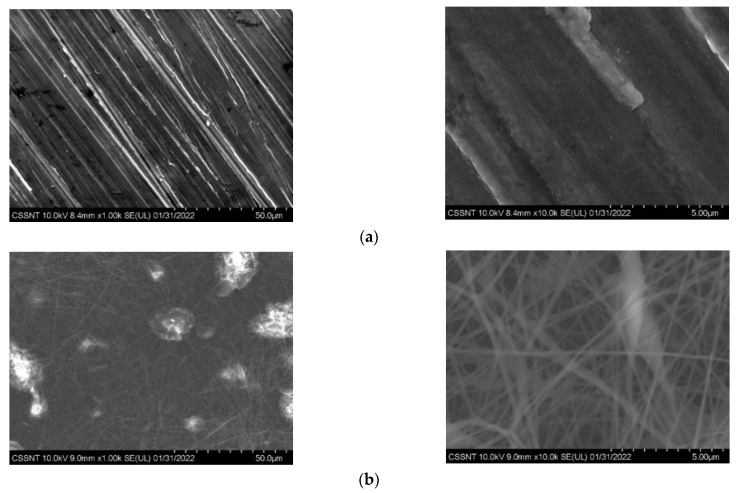
SEM micrographs of (**a**) uncoated Mg alloy, and (**b**) Mg alloy coated with PLA nanofibers at 1k and 10k magnifications, respectively.

The SEM micrographs of the uncoated Mg alloy (Figure 2a) show a typical morphology of a polished metal alloy, revealing stripe-like structures along the surface. In the PLA coated Mg alloy sample (Figure 2b), the nanofibers were clearly visible on the surface along with formations that appeared to be nodules of nanofibers. The distribution of the nanofibers was homogenous over the surface of the entire sample.

The energy dispersive X-ray spectroscopy analysis confirmed the presence of PLA and showed the same uniformity in the distribution of the PLA nanofibers. In the results obtained (Table 2) on the uncoated Mg alloy we observed that more than 85 wt% of the sample was composed of Mg (87.83 wt% ± 0.47 wt%), Al (2.62 wt% ± 0.10 wt%), and Zn (1.02 wt% ± 0.08 wt%). The composition was completed by C and O which are often contaminants in this situation. In the coated sample, C (58.54 wt% ± 1.82 wt%) and O (20.74 wt% ± 2.96 wt%) were the main identified elements. This corroborated with the FT-IR results showing that the sample was coated with PLA nanofibers. Furthermore, the presence of the Mg alloy as substrate could be seen in the results obtained on the coated Mg alloy, all three of its components being present (Mg, Al, and Zn). All the EDX results presented in Table 2 are averages of measurements recorded on four areas of each sample.

#### 3.1.3. Hydrophilicity and Hydrophobicity of Mg Alloy

One of the most frequently applied methods for determining a surface’s wettability is to measure the contact angle. Distilled water, ethylene glycol, and diiodomethane were the solvents used. The components of the surface free energy (SFE) properties of these solvents are given in Table 3 [30]. Contact angle measurement gives indications for surface wettability and are used for the determination of SFE. The average values of the contact angles of the coated and uncoated Mg alloys are summarized in Table 4.

The contact angle values for Mg alloy-PLA indicated that the coating resulted in increased hydrophobicity (>90), which leads to reduced platelet adsorption and subsequent thrombogenicity, as previously shown [31]. Research studies show that hydrophobic surfaces favor the sorption of a wide range of proteins such as albumin, fibronectin, IgG, etc. However, when fibrinogen is in contact with a hydrophobic substrate, it does not usually attach and will not trigger the thrombogenic cascade, therefore avoids blood clots from forming on the implant’s surface. Furthermore, hydrophobic surfaces are preferred by endothelial cells, giving the implant surfaces improved biocompatibility [32,33].

SFE has been determined as the sum of two independent contributions (Equation (4)) according to the Owen–Wendt method [30].

(4) $$\gamma_{s}=\gamma_{s}^{d}+\gamma_{s}^{p}$$

where $\gamma_{s}$ is the SFE, $\gamma_{s}^{d}$ is the dispersion component of SFE, and $\gamma_{s}^{p}$ is the polar component of SFE [30].

Although the calculations of SFE are based on the approximation of perfectly smooth surfaces that are almost impossible to generate, the determination of the SFE of samples covered with PLA electrospun nanofibers was calculated using the same method by others [34].

The SFE of Mg alloy and Mg alloy-PLA in our investigation was 45.01 mJ/m^2^ and 37.55 mJ/m^2^, respectively. The surface free energy decreased as a result of the PLA coating, which can be attributed to the interaction between the polar regions of the polymer and water. Furthermore, the obtained SFE values indicated that the material could present biocompatibility since they were in the range of 30–50 mJ/m^2^, in accordance with the literature [34].

For Mg alloy and Mg alloy-PLA, the fractional polarity, FP ($\gamma^{p}/\gamma^{p}$ + $\gamma^{d})$, was 0.52 and 0.04, respectively. It has been shown that an FP of less than 0.3 promotes cellular adhesion and fibroblast proliferation [35,36].

#### 3.1.4. Hydrogen Release in SBF

**Figure 3:**
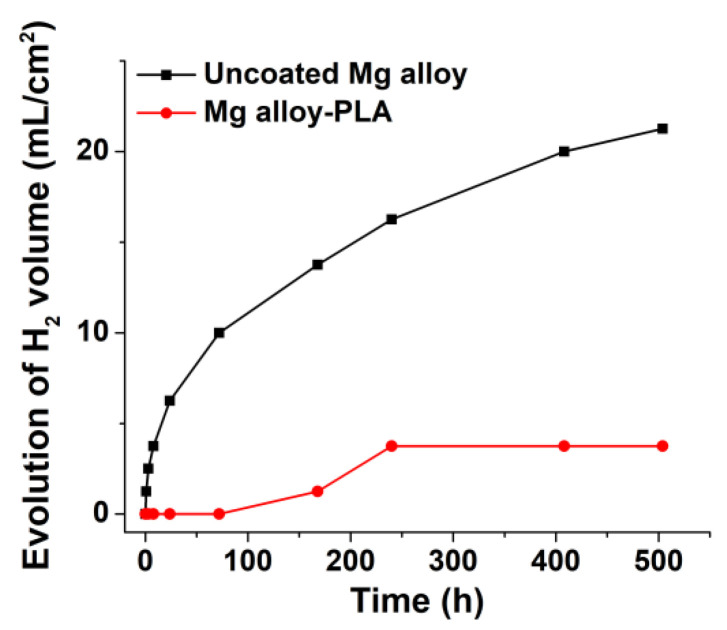
Evolution of H_2_ release rate of uncoated Mg alloy and Mg alloy-PLA samples in SBF.

The results of measuring the evolution of the H_2_ volume are shown in Figure 3 and were achieved by immersing the samples in SBF solution for up to 21 days to monitor the corrosion rates of the Mg alloy and Mg alloy-PLA nanofiber samples. Due to the negative electrochemical potential, hydrogen bubbles appeared as soon as the Mg samples were immersed in the SBF solution. It could be seen that uncoated Mg had a faster corrosion rate compared to Mg alloy-PLA, which showed no hydrogen release until day 7. In the first 72 h of immersion, the evolution of H_2_ was negligible for the coated sample. During this time, the SBF diffused through the PLA nanofiber coating, starting the corrosion of the Mg alloy beneath the polymeric coating. The resulting corrosion products remained between the PLA film and the metallic surface of the sample. After 72 h of immersion PLA degradation was sufficient to allow the produced H_2_ to be suddenly released from the surface of the sample. The evolution of H_2_ was observed until 240 h immersion time. During this time, the corrosion products continued to grow and the SBF salts started to accumulate on the sample surface. After 240 h of immersion, the H_2_ release slowed down due to the accumulation of corrosion products and alkalinization of the environment, as well as the increase of the thickness of the SFB salt deposits.

Therefore, the uncoated Mg alloy, after 504 h of immersion, had a total volume of H_2_ released of 21.25 mL/cm^2^, compared to the Mg alloy-PLA, which had a total volume of H_2_ of 3.75 mL/cm^2^. The H_2_ release rate was 1.01 mL/cm^2^/day and 0.18 mL/cm^2^/day for Mg-alloy and Mg alloy-PLA, respectively. According to reports, the human body can sustain a hydrogen adsorption rate of 2.25 mL/cm^2^/day [37].

The corrosion rate was calculated using Equation (2), from the volume of H_2_, and the result on the uncoated Mg alloy was 2.03 mm/year, while on Mg alloy-PLA it was 0.36 mm/year.

#### 3.1.5. pH Variation

**Figure 4:**
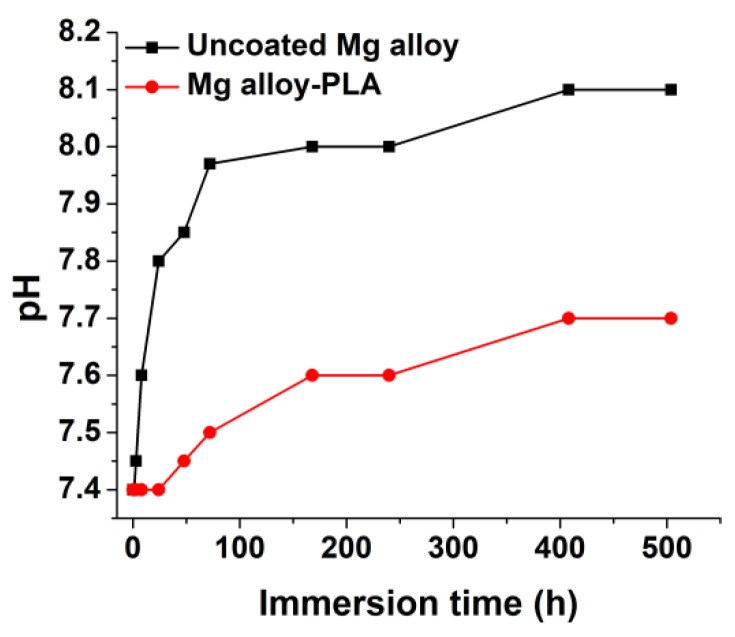
Evolution of the pH of the samples of uncoated Mg alloy and Mg-PLA, in SBF.

Measuring the pH variation is important in the evaluation of the corrosion process. At the time of the immersion of the uncoated Mg alloy sample and the Mg alloy-PLA in SBF, the degradation process of the uncoated sample began immediately, as can be seen in Figure 4. Calcium phosphates were generated and deposited on the surface of the alloy over time [38], slowing the rate of corrosion and making the environment around the implant alkaline [39].

After 3 h of immersion, the pH of the SBF solution increased. The development of a protective layer of Mg(OH)_2_ on the surface of the Mg alloy explains this enhancement. The pH of the Mg alloy-PLA increased only after 48 h, which was mainly due to the deterioration of the coating and ion penetration into the Mg substrate.

#### 3.1.6. Electrochemical Impedance Spectroscopy (EIS) and Potentiodynamic Voltammetry (Tafel Curves)

**Figure 5:**
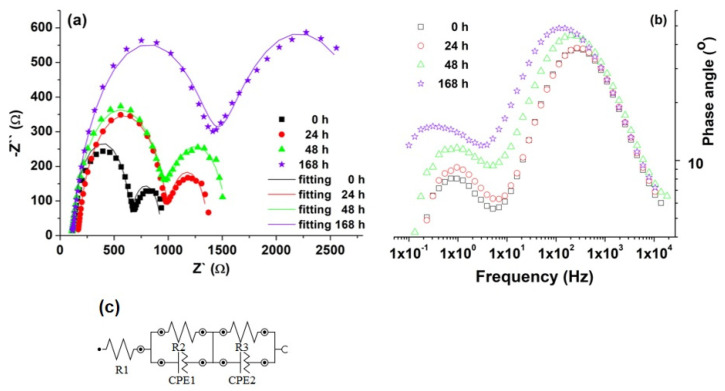
Nyquist (**a**), Bode (**b**) diagrams, and (**c**) equivalent circuit from 0–168 h of uncoated Mg alloy, immersed in SBF.

**Figure 6:**
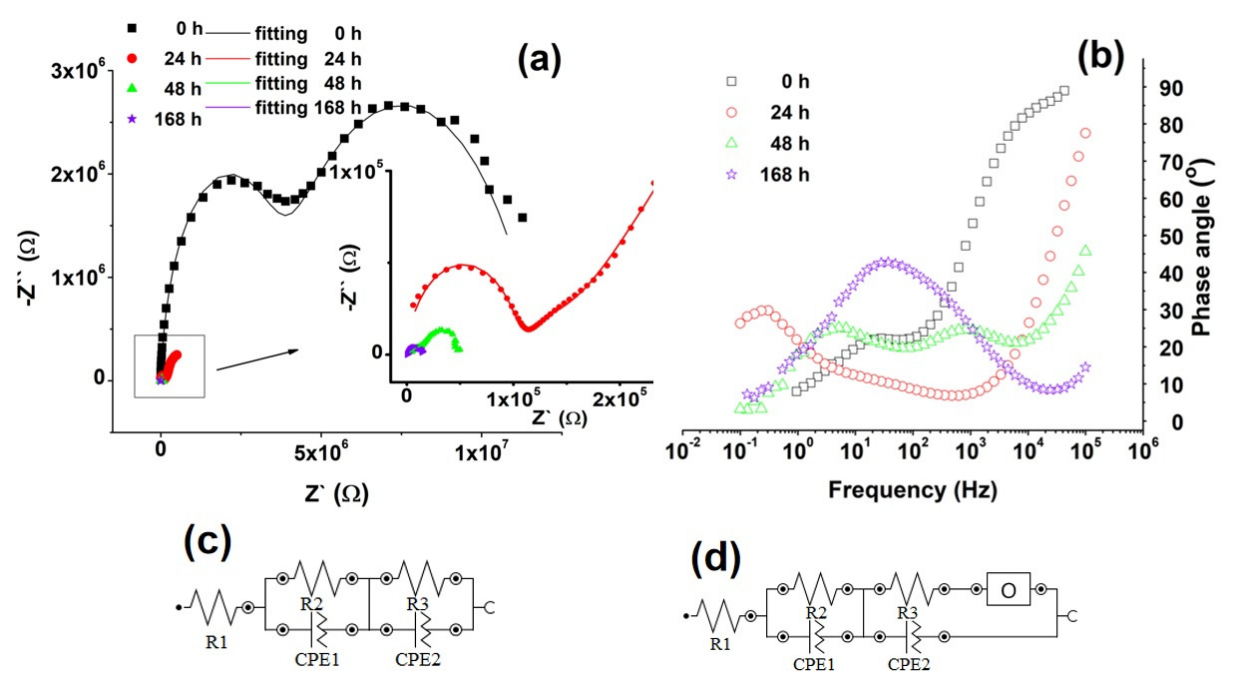
Nyquist (**a**), Bode (**b**) diagrams, and (**c**) equivalent circuit from 0 h, (**d**) equivalent circuit from 24–168 h of Mg alloy-PLA, immersed in SBF, over time.

Figure 5 and Figure 6 represent the Nyquist, Bode diagrams, and the equivalent circuits of uncoated Mg alloy and Mg alloy-PLA, at different periods of immersion time in SBF.

The Nyquist plots of the Mg alloy exhibited, during the entire immersion time in SBF solution, two well-defined loops with different diameters, which indicated the same corrosion mechanism but different corrosion rates [40]. The Bode phase diagram shows that the phase angle moved at lower frequencies and increased slightly with the immersion time. This is a consequence of the accumulation of corrosion products on the alloy surface which leads to an increased mass or thickness which could provide a certain protective property [41].

The equivalent circuit and the corresponding electrochemical parameters used to fit the impedance data are shown in Figure 5c, and Table 5. The elements in the equivalent circuit were attributed as follows: At 0 h, R1 is the resistance of the electrolyte, the parallel (R2/CPE1) is the interface between the electrolyte and the initial oxide film, the parallel (R3/CPE2) is the interface between the initial oxide layer and the metallic substrate. At 24 h, 48 h, and 168 h, R1 remained attributed to the electrolyte resistance, the interfaces represented as the parallel (R2/CPE1) and (R3/CPE2) now incorporated besides the initial oxides, the corrosion products and salts resulting from the immersion in SBF. The use of a constant phase element is considered to be the electrical double layer due to the inhomogeneous electrode surface.

From the EIS spectra, we can deduce that at 0 h the Mg alloy was covered with thin oxides produced naturally in the atmosphere. After immersion in SBF, during the observed period, a few competing processes occurred on the surfaces of the samples, namely the oxidation of the alloy, the deposition of salts from the electrolyte, the release of H_2_, which partially destroyed the obtained oxide and salt films. The corrosion resistance, in this case, was given not by the passivating processes but by the lower conductivity of the salt depositions.

At 0 h, we could see two capacitive loops in the Mg alloy-PLA, as shown in Figure 6a. A small capacitive loop at a high frequency area could be attributed to the coating, whereas a large capacitive loop at a medium frequency region may be attributed to electrochemical processes occurring below the coating [42]. This indicates that in the SBF solution, the Mg alloy was only partially protected.

This phenomenon is due to the PLA network that allows the SBF solution to penetrate. At 24 and 48 h, the diffusion of SFB was evidenced by the EIS. After 24 h of immersion the diffusion of SFB was evidenced by the EIS. During the immersion, on the electrode surface, below the PLA nanofiber layer, corrosion products started to accumulate. During the corrosion process, the dissolved oxygen as a depolarizing agent needs to go through the corrosion product layer before reaching the electrode reaction interface. The diffusion of cathode depolarizing agent through the corrosion product layer is shown as cotangent-hyperbolic diffusion impedance (O) in the impedance response. In this condition, for Mg alloy-PLA, two different electrochemical equivalent circuit models are proposed. As a result, we can see R1 as the solution resistance, parallel R2/CPE1 as the electrolyte-PLA interface, and parallel linked elements (R3-CPE2) as the electrical charge transfer at the Mg alloy substrate/PLA coating interface in Figure 6c,d and Table 6. A cotangent-hyperbolic diffusion impedance (O) was added in series with R3 to improve the fit over 24–168 h. This cotangent-hyperbolic diffusion impedance (O) is used to model limited length diffusion and investigate how reactants diffuse through permeable defects [43,44].

**Figure 7:**
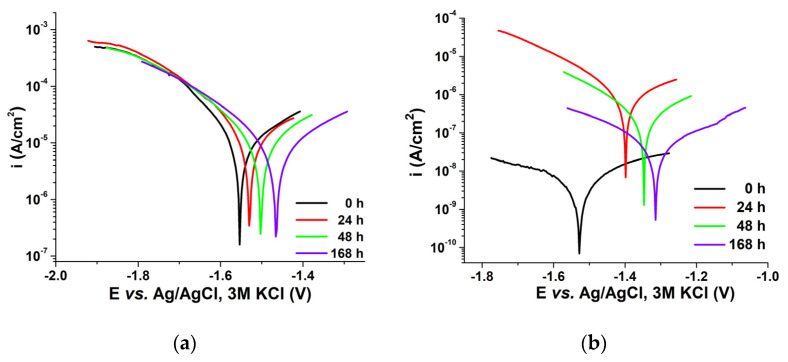
Tafel curves for (**a**) uncoated Mg alloy, and (**b**) Mg alloy-PLA, immersed in SBF, over time.

According to Figure 7 and Table 7, at 0 h, the corrosion rate (V_corr_) in the case of Mg-PLA was 0.048 μm/year and, after 24 h, an increase in the corrosion rate was observed, due to the penetration of ions from the electrolyte to the Mg substrate. Therefore, a shift of the corrosion potential towards more electropositive values can be observed with the increase of the immersion time.

Furthermore, comparing the corrosion current density (I_corr_) we observed much lower values for Mg alloy coated with PLA nanofibers than uncoated Mg alloy. This indicates better corrosion resistance by the coated material [45,46].

Since electrochemical stability is a function of hydrogen evolution, pH and surface energy changes, the interaction of the Mg alloy with the PLA electrospun nanofiber coating involves the changes of corrosion, wettability, and pH, as were described above. All of them allowed us to propose an empiric model with three stages.

The first is hydrogen evolution, the second is Mg hydroxide dissolution and the third is the increase of hydrophobic character with the decrease of adherence.

**Scheme 2:**
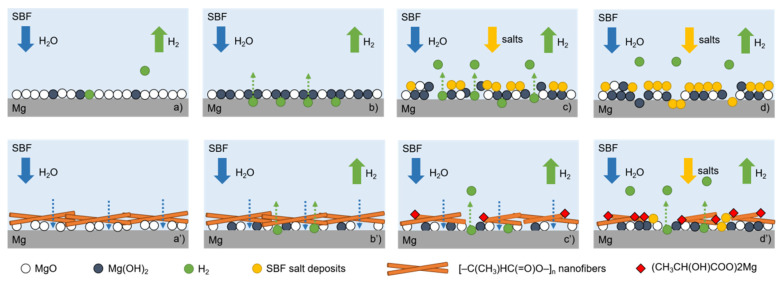
The empiric model of Mg alloy interaction with PLA nanofibers.

The main processes represented in the above model (Scheme 2) for biodegradable Mg alloy with and without PLA nanofibers are described as follows:

Uncoated sample:

(a) Uncoated sample immersion in SBF: the presence of natural Mg oxides formed in the atmosphere.
(b) Mg(OH)_2_ and H_2_ formation.
(c) H_2_ destroyed MgO and Mg(OH)_2_ films; deposit of salts from SBF.
(d) MgO, Mg(OH)_2_, and salts covered the Mg surface; due to H_2_ release the film had cracks.

Coated sample:

(a’) Sample coated with PLA nanofibers in SBF: the presence of natural Mg oxides formed in atmosphere under PLA; SBF diffusion through nanofibers.
(b’) Mg(OH)_2_ and H_2_ formation.
(c’) PLA nanofibers degradation; (RCOO)_2_ Mg formation; H_2_ broke the polymeric film.
(d’) Deposition of salts from SBF will occupy free spaces without PLA.

As a result of the interaction with PLA nanofibers, as has been seen from the contact angle determinations, the coated sample had a hydrophobic character and higher surface energy than the uncoated one.

## 4. Conclusions

The paper is a study of the synthesis and characterization of Mg alloy coatings conducted to enhance the potential applications of PLA nanofibers on biodegradable Mg alloy. The procedure of synthesis was an electrospinning technique, and the deposition was evidenced using scanning electronic microscopy with EDX for elemental composition and FT-IR. Both confirmed the successful deposition of the PLA nanofibers on the Mg alloy. The degradation tests of the coated and uncoated alloys were performed in SBF. Based on the experimental data, it can be concluded that electrospun PLA nanofibers improve the surface behavior, having a higher electrochemical stability in SBF, a lower volume of hydrogen release, and an important increase of the hydrophobicity characteristics.

Taking into account corrosion rate, wettability, and pH changes, an empiric model of the interaction of an Mg alloy with PLA nanofibers was proposed. The main chemical reactions which sustain the model define three stages, hydrogen evolution, Mg hydroxide dissolution, and the increase of hydrophobic character with the decrease of adherence. Based on such behavior, biodegradable Mg alloys with PLA electrospun nanofibers could be a good proposal for other biomedical applications, such as platelet adhesion decrease in thrombosis or behavior in drug release.

## Figures and Tables

**Table 1:** Composition of the simulated body fluid (SBF) [24].

Component	Concentration [g/500 mL]
NaCl	3.998
NaHCO_3_	0.175
KCl	0.112
K_2_HPO_4_·3H_2_O	0.114
MgCl_2_·6H_2_O	0.152
CaCl_2_	0.139
Na_2_SO_4_	0.035
(CH_2_OH)_3_CNH_2_	3.028

**Table 2:** Elemental composition in wt% (obtained by EDX) of the uncoated and coated Mg alloy.

Element	Uncoated Mg Alloy		Mg Alloy-PLA	
	Wt%	Std. Dev.	Wt%	Std. Dev.
Mg	87.83	0.47	19.81	3.15
Al	2.62	0.1	0.67	0.14
Zn	1.02	0.08	0.16	0.16
C	3.83	0.30	58.54	1.82
O	4.59	0.31	20.74	2.96

**Table 3:** Surface free energy of the liquids at 20 °C [30].

Liquids	Surface Energy [mJ/m^2^]		
	$\gamma_{s}$	$\gamma_{s}^{d}$	$\gamma_{s}^{p}$
Water	72.8	21.8	51
Ethylene glycol	47.7	21.3	26.4
Diiodomethane	50.8	48.5	2.3

**Table 4:** Values of contact angle, and surface free energy.

	Uncoated Mg Alloy	Mg Alloy-PLA
Water, °	59 ± 0.5	98 ± 0.3
Ethylene glycol, °	44 ± 0.2	72 ± 0.6
Diiodomethane, °	57 ± 0.6	62 ± 0.4
$\gamma_{s}$ (SFE), mJ/m^2^	45.01	37.55
$\gamma_{s}^{p}$, mJ/m^2^	23.74	1.59
$\gamma_{s}^{d}$, mJ/m^2^	21.27	35.96

**Table 5:** Values from equivalent circuit uncoated Mg alloy.

Sample	Time [h]	R1 [kΩ]	R2 [kΩ]	CPE1		R3 [kΩ]	CPE2	
				Y_O_ [μMho]	N1 *		Y_O_ [μMho]	N2 *
Uncoated Mg alloy	0	0.117	0.576	5.46	0.881	0.273	874	0.964
	24	0.116	0.837	4.02	0.877	0.365	672	0.967
	48	0.110	0.881	6.16	0.866	0.563	563	0.908
	168	0.115	1.32	7.71	0.855	1.61	531	0.783

* N1, N2 index that represents the deviation from a pure capacitor of CPE.

**Table 6:** Values from equivalents circuits Mg alloy-PLA.

Sample	Time [h]	R1 [kΩ]	R2 [kΩ]	CPE1		R3 [kΩ]	CPE2		O	
				Y_O_ [μMho]	N1 *		Y_O_ [μMho]	N2 *	Y_O_ [μMho]	B
Mg alloy-PLA	0	0.115	3390	69.6 × 10^−5^	0.982	8460	6.46 × 10^−3^	0.705	-	-
	24	0.113	46.6	0.659	0.566	101	1.06 × 10^−4^	0.961	3.23	1.88
	48	0.11	2.62	3.23 × 10^−3^	0.895	14.7	1.43	0.547	12.5	0.407
	168	0.119	0.324	1.61 × 10^−3^	0.9	5.88	9.86	0.633	10.4	0.1

* N1, N2 index that represents the deviation from a pure capacitor of CPE.

**Table 7:** Tafel parameters for samples of uncoated Mg alloy, and Mg-PLA alloy.

Sample	Time [h]	E_corr_ [V]	I_corr_ [μA]	V_corr_ [μm/Year]	B_a_ [V/Decade]	B_c_ [V/Decade]
Uncoated Mg alloy	0	−1.55	26.8	2114	0.19	11.33
	24	−1.53	22.3	1756	0.19	0.60
	48	−1.50	17.05	1344	0.22	0.37
	168	−1.47	2.38	187.58	0.08	0.07
Mg alloy-PLA	0	−1.52	0.0021	0.048	0.20	0.14
	24	−1.31	0.0195	0.45	0.15	0.23
	48	−1.35	0.126	2.91	0.25	0.34
	168	−1.39	0.382	8.82	0.23	0.25

## Data Availability

Not applicable.
